# Real-time microstructure imaging by Laue microdiffraction: A sample application in laser 3D printed Ni-based superalloys

**DOI:** 10.1038/srep28144

**Published:** 2016-06-15

**Authors:** Guangni Zhou, Wenxin Zhu, Hao Shen, Yao Li, Anfeng Zhang, Nobumichi Tamura, Kai Chen

**Affiliations:** 1 Center for Advancing Materials Performance from the Nanoscale (CAMP-Nano), State Key Laboratory for Mechanical Behavior of Materials, Xi’an Jiaotong University, Xi’an, Shaanxi 710049, P.R. China; 2 State Key Laboratory for Manufacturing Systems Engineering, Xi’an Jiaotong University, Xi’an, Shaanxi 710049, P.R. China; 3 Advanced Light Source, Lawrence Berkeley National Laboratory, Berkeley, California 94720, USA

## Abstract

Synchrotron-based Laue microdiffraction has been widely applied to characterize the local crystal structure, orientation, and defects of inhomogeneous polycrystalline solids by raster scanning them under a micro/nano focused polychromatic X-ray probe. In a typical experiment, a large number of Laue diffraction patterns are collected, requiring novel data reduction and analysis approaches, especially for researchers who do not have access to fast parallel computing capabilities. In this article, a novel approach is developed by plotting the distributions of the average recorded intensity and the average filtered intensity of the Laue patterns. Visualization of the characteristic microstructural features is realized in real time during data collection. As an example, this method is applied to image key features such as microcracks, carbides, heat affected zone, and dendrites in a laser assisted 3D printed Ni-based superalloy, at a speed much faster than data collection. Such analytical approach remains valid for a wide range of crystalline solids, and therefore extends the application range of the Laue microdiffraction technique to problems where real-time decision-making during experiment is crucial (for instance time-resolved non-reversible experiments).

Laue microdiffraction (μXRD) using micro-focused high-intensity polychromatic X-ray beam obtained at synchrotron facilities, has found applications in a wide range of scientific disciplines such as materials, geological, and environmental sciences. These studies usually involve two-dimensional raster scanning of an area of the sample, with recording of a Laue diffraction pattern at each scanning position. With this technique, minute amounts of crystals in heterogeneous matrices can be identified^1^. Phase transformation can be detected at micro/nano scale^2^, local crystal orientation and thus crystal grain boundaries mapped^3^, elastic strain tensors measured at the subgranular scale^4^, and plastic deformation around a crack tip investigated^5^. Albeit the large potential of materials characterization capabilities, μXRD data analysis is non-trivial. In a typical μXRD data analysis procedure^6^, all the Laue patterns are indexed with one or several possible crystal structures to obtain the local crystal orientation, and then the positions of all the indexed Laue diffraction peaks in each pattern are compared with the calculated ones to derive the deviatoric lattice strain tensor. Laue peak intensity is also taken into account for speeding the indexation process, unequivocally indexing trigonal crystal diffraction patterns^7^,^8^, and refining small molecule crystal structure^9^. These approaches usually take longer than the data collection for researchers who do not have access to fast parallel computing capabilities. It is expected that this issue will become even more serious with the advent of nano-beam X-ray Laue diffraction achieving better spatial resolution^10^, and for materials systems that are extremely non-uniform. Here, we develop a novel approach which takes the diffraction and background intensity into account to achieve rapid microstructural imaging. With this approach, the data analysis is greatly accelerated and can be easily accomplished on a regular PC in real time as the μXRD scan is conducted.

To demonstrate this newly developed method, we take the laser assisted 3D printed Ni-based superalloy as an example. Laser 3D printing is considered to be one of the most promising refurbishing technologies for extending the service lifetime and reducing overall cost of directionally solidified Ni-based superalloy blades and blisks^11^,^12^. Albeit its unique advantages as a refurbishing technology, 3D printing is also facing some problems related to microstructural defects. For example, hot cracking happens in the laser heating induced heat affected zones (HAZs)^13^,^14^. The introduction of carbone leads to the formation of a variety of carbides, such as M_23_C_6_, M_6_C, and MC^15^, which could impose significant influence on the mechanical properties of Ni-based superalloy^16^,^17^. What is more, the crystal orientation of the substrate can be preserved in the laser cladding layers, while the dendrites in the epitaxial zone are usually much finer than the traditionally cast alloys due to the high thermal gradient and fast solidification rate^18^. Previous studies also reveals changes in microstructural inhomogeneity from the center to the edge of dendrites^19^,^20^. It is therefore essential to investigate the size, shape, microstrain/microstress, and defect density of the epitaxial dendrites at the sub-dendritic micrometric level.

In previous studies, the crystal orientation has been characterized using electron backscatter diffraction (EBSD) on various Ni-based superalloys^21^,^22^. High resolution X-ray diffraction (HRXRD) and reciprocal space mapping (RSM) have also been employed to study the disorientation and lattice mismatch of the laser deposited layers and the substrate^23^,^24^. The size, morphology, and crystal orientation of the carbides have been investigated using scanning/transmission electron microscope (SEM/TEM)^25^. The initiation of cracks has been studied by combining serial sectioning, EBSD and quantitative fractographic analysis^26^. In recent literatures, μXRD has been employed to map the distribution of defect density and subgrain misorientation in the epitaxial region, and to unveil the mechanism of the columnar-to-equiaxed transition (CET) in 3D printed Ni-based superalloys^19^,^20^. However, there is no report of an experiment that characterizes all these key features of 3D printed Ni-based superalloy simultaneously. In this article, we demonstrate that μXRD, combined with advanced data analysis approach, can investigate almost all the microstructures mentioned above in real time on a personal laptop as the diffraction data are collected.

## Results

### Generation of microstructure maps

The 3D printed Ni-based superalloy specimen is mounted on the 2D scanning stage on the μXRD beamline 12.3.2 at the Advanced Light Source with its epitaxial dendrite growth direction aligned along the scanning **Y**-direction. An area of 130 μm in width (**X**-direction) and 408 μm in length (**Y**-direction) across the substrate-cladding interface is scanned with 2 μm step size using the μXRD technique and 13260 Laue patterns (65 × 204) are recorded.

To analyze the μXRD data, three greyscale maps, each with 65 × 204 pixels, are generated (brighter color represents higher intensity), corresponding to the size of the μXRD scan. In this article, when referring to a position in the maps, we use the term “spot”, while the term “pixel” refers to a position in the Laue pattern. For convenience, the value of the *k*th pixel in the *i*th spot of the Laue scan is denoted as 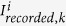. The value of the *i*th spot in the first map (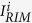) is equal to the average intensity of all the pixels in the *i*th Laue pattern without any treatment, and thus it is called the “Recorded Intensity Map” (RIM) in this study. Mathematically, this procedure can be simply expressed as:


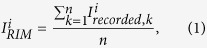


where *n* is the number of pixels in a Laue pattern.

The second map, the “Background Intensity Map” (BIM), is created following the method developed and implanted in the software package XMAS^6^. In this approach, the background of each Laue pattern is fitted using a two-dimensional generalization of the Brückner algorithm^27^, which was originally developed for one dimensional powder diffractograms.

In the third map called “Filtered Intensity Map” (FIM), we show the average intensity of each Laue pattern after a filter has been applied. In the computer program we developed, two options for the filter can be selected. Option 1 is using the background intensity that is calculated from BIM, and thus the value of the *i*th spot in this map is computed as


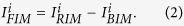


Option 2, adopted in this article, defines a threshold value (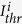) for the *i*th Laue pattern. The selection of the threshold influences the appearance of FIM. In this study, the threshold is defined as 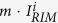, where *m* is a constant. It is evident that this threshold varies from spot to spot on the map. Based on our experience, the FIM appears the most informative contrast-wise when *m* is set to be 5 for most metals and minerals, and that it should be turned lower if the crystallinity of the sample is poor. The filtered detector pixel intensity (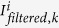) is counted as zero if the recorded intensity 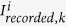 is less than or equal to the threshold value, while the treated pixel intensity is taken as the difference between the recorded intensity and the threshold value if 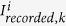 exceeds the threshold value:


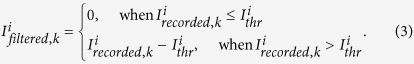


The intensities of all the pixels in a Laue pattern are averaged to obtain the value of the corresponding spot in the FIM:


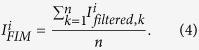


With either option, not only the background, but also a certain proportion of the diffraction peaks is subtracted from the Laue patterns.

### Interpretation of the microstructure maps

**Figure 1:**
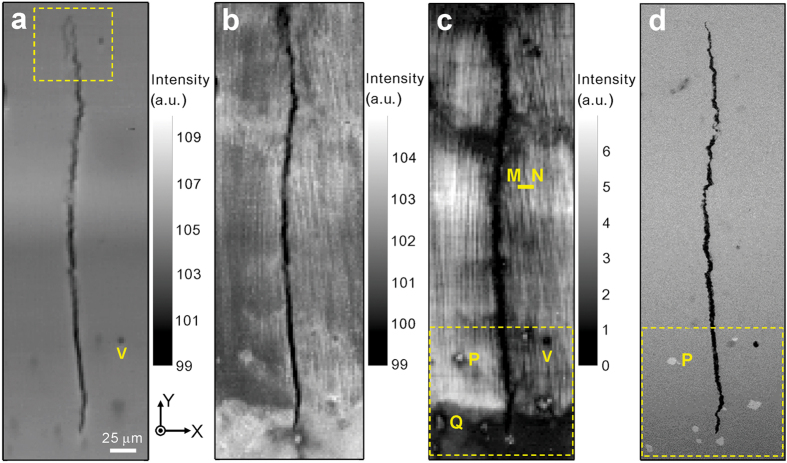
Characterization of the microstructural features. (**a**) Recorded Intensity Map (RIM), (**b**) Background Intensity Map (BIM), (**c**) Filtered Intensity Map (FIM) obtained from μXRD scan and (**d**) BSE-SEM image display the same area across the cladding-substrate interface of Ni-based superalloy, where a horizontal interface, a vertical crack, micro-sized precipitates are observed.

In the RIM displayed in Fig. 1a, a crack, about 6 μm (3 pixels) wide, is visible and roughly parallel to the **Y**-direction. Although the detailed mechanism is beyond the scope of this article, it is believed that the crack is formed during the 3D printing process due to the local residual strain and materials deformation. Some dark particulates are also observed in the RIM, most of them distributed in the region that is within about 100 μm from the bottom. In the BIM shown in Fig. 1b, the crack shows similar contrast and width to in the RIM, except that the tip appears much fuzzier. High intensity and vertical strip-like features are visible at the bottom and upper regions of the map, respectively. In the FIM shown in Fig. 1c, the vertical crack in the middle of the image appears much wider than in Fig. 1a,b, and the edge of the crack is less sharp. Most of the particulates, except the much darker one labeled as V in Fig. 1a,c, show higher intensity than their adjacent region. An interface is apparent along the **X**-direction about 40–50 μm from the bottom of the scanned area. The region below the interface appears much darker than the one above it. In the brighter region above the interface, vertical strip-like features are observed with better contrast and resolution than in Fig. 1b. The width of most of the strips is approximately 4–6 μm. Although almost all the features in BIM can be seen in either RIM or FIM in this case, we want to emphasize that BIM usually provides important hints to understand the features observed in RIM or FIM. The FIM plotted following Option 1 is shown in Fig. S1, which looks almost identical to Fig. 1c. The differences between the maps obtained with these two options are discussed in Section 2 of the supplementary information.

By comparing the RIM, FIM, and the SEM image taken under the back-scattered electron (BSE) mode of the same sample region (Fig. 1d), it is found that the imaging resolution of the crack is similar in the RIM and BSE image, but better than in the FIM. It is also confirmed that almost all the particulates are precipitates with different chemical composition from the matrix, except the one labeled as V, which is a void formed during the 3D printing process. The contrast of these precipitates in the FIM is much better than that in the RIM. From the BSE image with the magnification as shown in Fig. 1d, it is difficult to tell what the strip-like features are in the FIM. The interpretation of these strip-like features is given in the next section.

## Discussion

The intensity recorded by the detector includes two main contributions: the diffraction and fluorescence signals from the crystals irradiated by the incident polychromatic X-ray beam. From our description, it is clear that the value at a certain spot in the RIM represents the summation of all these signals from the irradiated volume of the specimen. On the other hand, our specimen is in first approximation chemically uniform except in the precipitates, voids, and cracks, and our μXRD experiment is carried out at a fixed geometry. It is therefore expected that the summation of the integrated intensities of all diffraction peaks plus the fluorescence signals from the specimen would be approximately uniform in the scanned region. This is why features such as precipitates, voids and cracks, and only these features, are visible in the RIM, and the imaging resolution of these features are only limited by the largest value between the scanning step size, X-ray beam size, and the interaction volume of the X-ray beam with the specimen. In other words, better imaging resolution will be achieved in the RIM if finer scanning step size is used, smaller X-ray beam size is available, and for samples with higher atomic number and mass density.

In contrast to the RIM, the value of a spot in the FIM is almost solely composed of diffraction signals. It is worth noting that in this study we define a fixed threshold to filter the detector recorded intensity, instead of the more conventional approach where the threshold is defined as the background level fitted by a polynomial function. As a result, not only fluorescence signals are filtered, but part of the intensity from the diffraction peaks is also subtracted, as if the peaks are chopped off at the root. If we assume that the total diffracted intensity of all the Laue peaks from the matrix is constant, one could expect that the images with sharper peaks would have less counts subtracted and more intensity retained by our filter.

**Figure 2:**
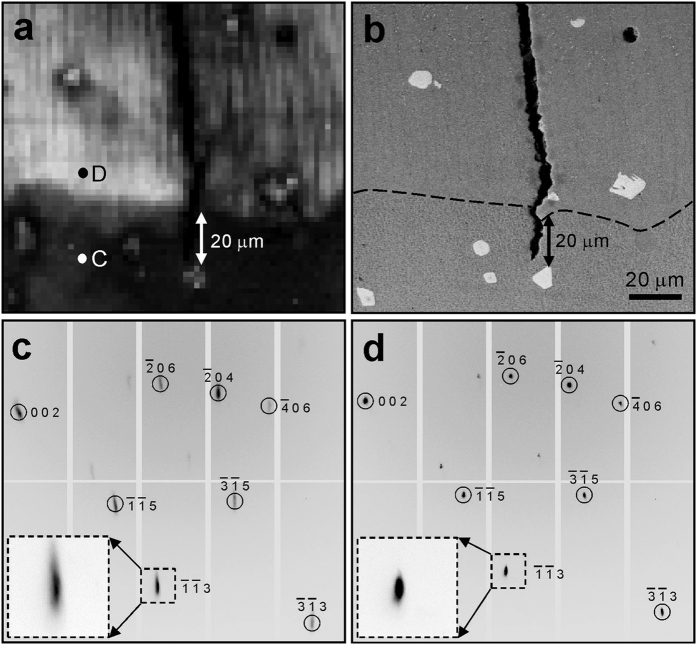
Low intensity at the bottom of FIM. (**a**) Magnified FIM and (**b**) BSE-SEM image suggest that the low intensity in the FIM represents the heat affected zone (HAZ) in the substrate. (**c**) Peak streaking is observed in the Laue diffraction pattern from the HAZ, suggesting high density of defects. (**d**) The peaks are sharp in the Laue pattern taken from the cladding layers.

To explain why a boundary is observed at the bottom of the FIM, the same region indicated by the yellow dashed squares in the FIM and the BSE image is magnified and shown in Fig. 2a,b, respectively. The distance from the boundary to the precipitate near the root of the crack is measured to be 20 μm in the FIM, and found to be identical to the distance from the substrate-cladding interface to the same precipitate in the BSE image. Two Laue patterns are selected along a vertical line, one in the dark region (Fig. 2c), and the other from the bright region (Fig. 2d). The diffraction peaks in Fig. 2c are much more smeared than the ones in Fig. 2d, indicating high density of dislocations in the substrate right below the interface, which is the so-called heat affected zone (HAZ)^28^. It is noted that the Laue peaks in these two patterns appear at similar positions on the detector, indicating the crystal orientation is retained from the substrate to the laser cladding layers.

**Figure 3:**
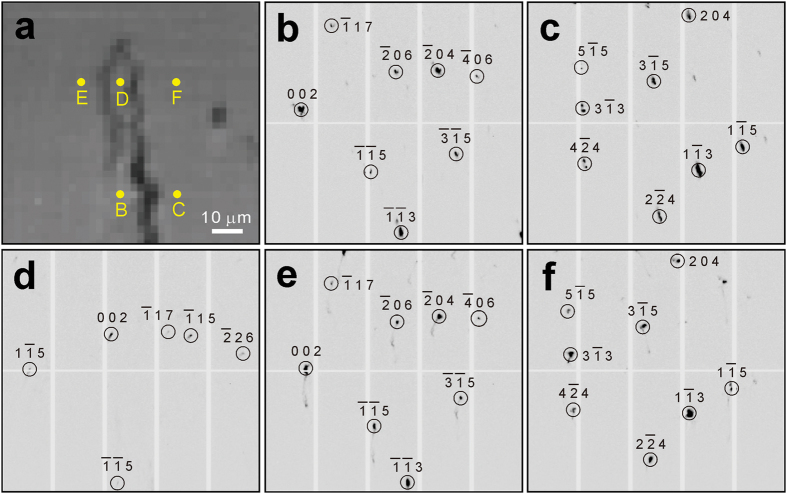
Characterization of the crack tip. (**a**) Magnified RIM of the crack tip area, marked by the box in Fig. 1a. (**b**–**f**) Diffraction patterns taken in typical regions marked as B to F in (**a**), suggesting that the crack propagates along high angle grain boundary.

The crack appears clearly with sharp edges in the RIM but fuzzy in the FIM, because the materials close to the crack are deformed plastically, and the Laue peaks taken in this region are broadened, which results in lower intensity values in the FIM than those less plastically deformed regions away from the crack. Comparing the morphology of the crack from the various images, it is found that the crack tip looks sharp and straight in the BSE image, but a hook appears at the tip in the RIM. We attribute this significant difference to the different probing depth between the two techniques. BSE signals are collected within only hundreds of nanometers under the sample surface, while the X-ray signals penetrate tens of microns deep into the sample. The hook-shaped crack tip is therefore a feature buried under the sample surface. To understand why the crack propagates along such route, we enlarged the sample region close to the crack tip, and five patterns are selected from that region and indexed (labeled in Fig. 3a). It is found that in most area of this region, two orientations, which are epitaxial with the pair of grains in the substrate, are indexed. The corresponding Laue patterns with hkl Miller indices are shown in Fig. 3b,c. The crack propagates along the grain boundary between these pair of grains. However, a third orientation emerges, as shown in Fig. 3d, between the two existing grains without much influencing the orientation of these two grains (Fig. 3e,f), and thus two grain boundaries are formed in this region. Based on this observation, we propose that high thermal stress exists during the 3D printing process, concentrates at the grain boundaries and exceeds the yield strength of the material, and thus plastic deformation occurs. Cracks therefore tend to form and propagate along high angle grain boundaries. As regards to why the third orientation emerges, more study is needed. It may have formed during the rapid solidification process, but it is also possible that it grew from deep inside the bulk of the specimen.

**Figure 4:**
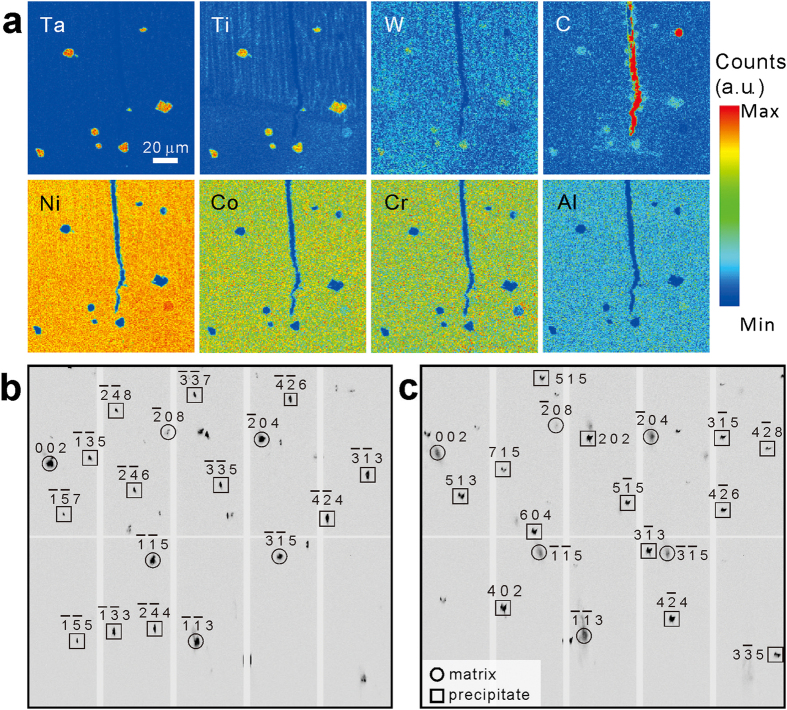
Characterization of carbide precipitates. (**a**) WDS mapping of the selected element indicates the precipitates are Ta, Ti, W, and C enriched. (**b**,**c**) Laue patterns of the precipitates, marked as P and Q in Fig. 1c, respectively, are indexed as NaCl structure, suggesting they are MC carbides. Q is invisible in the BSE-SEM in Fig. 1d, because it is buried under the Ni-matrix, beyond the probe depth of electron beam.

It is interesting to ask why the precipitates appear darker in the RIM but brighter in the FIM. According to the wavelength-dispersive X-ray spectroscopy (WDS) results, the precipitates are rich in the elements Ta, Ti, W, and C (Fig. 4a). The extremely high content of C inside the crack and the void is attributed to the use of SiC polishing paper and diamond polishing slurry during the cross-section specimen preparation. It is noted that the X-ray spectra employed in this study is 5–24 keV, which is not high enough to generate the strong fluorescence K lines for the high-atomic-number elements Ta and W. Therefore the overall detector recorded intensity of the precipitates is low, and these spots are darker in the RIM. To explain why the precipitates appear brighter in the FIM, we look closely at the Laue patterns taken on these spots, for instance the P position shown in Fig. 4b. Two sets of diffraction peaks can be indexed, one belonging to the Ni-based superalloy (FCC) matrix and the other belonging to a NaCl structure. From the WDS elemental analysis, the μXRD results, and combining with previous literature results^29^,^30^, these precipitates are identified as MC carbides (M = Ta, Ti, and W). Since FIM mainly sees diffraction signals, and heavy elements and sharp peaks give stronger intensity signal per pixel, precipitates appear brighter there. We notice that the Q particulate shows similar morphology and contrast as P in the FIM, but in BSE image and WDS scans nothing can be detected at this position. Analysis of the Laue pattern at this position (Fig. 4c) shows that a crystal grain with NaCl structure can be indexed besides a grain with FCC Ni structure, which indicates that very probably a MC carbide precipitate is buried here, beyond the probing depth of the electron beam used in SEM and WDS techniques. It is worth mentioning that a previous study has reported orientation relationships between the Ni-matrix and the MC carbides^31^, as {001}_carbide_//{001}_matrix_ and 〈001〉_carbide_//〈001〉_matrix_, but these relationships are not observed here. Therefore, more efforts are necessary to study how these carbide precipitates could influence the mechanical behaviors of the Ni-based superalloy.

**Figure 5:**
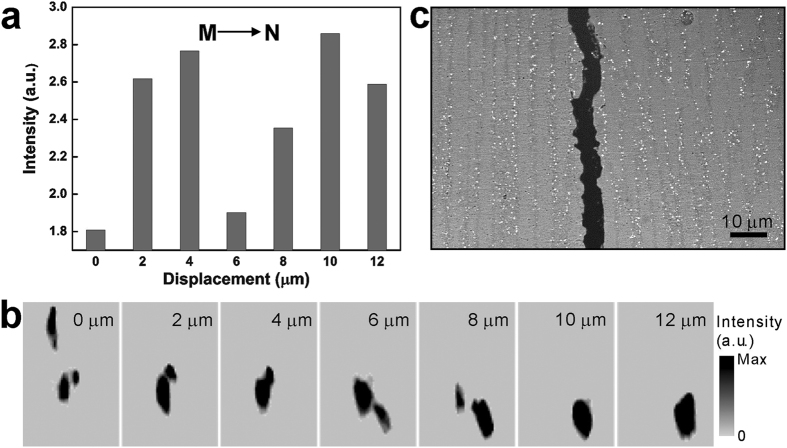
Characterization of interdendrite subgrain boundaries. (**a**) Spot-by-spot plotting of the filtered intensity along M-N shows a variation by about 50%. (**b**) Enlarged areas near the 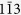 reflection on the Laue patterns indicate slight orientation change across the low angle inter-dendrite subgrain boundaries. (**c**) High-magnification BSE-SEM image shows inter-dendrite boundaries.

To interpret the vertical strip-like features visible in the FIM, Laue diffraction patterns across the strip boundaries are analyzed. The intensity histogram along the horizontal line labeled MN in Fig. 1c, is shown in Fig. 5a. It appears that the filtered intensity varies more than 50% along this line. The 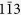 reflections of the Laue patterns taken along this line are shown in Fig. 5b. The crystal orientation along this line is almost constant, with relative rotation less than 1°, and thus the reflection position does not change much. However, the Laue diffraction peak shape alters significantly in conjunction with the intensity fluctuation. Peak splitting and therefore subpeaks are observed to correspond to the low intensity spots, while relatively sharp peaks are recorded at the spots with high intensity. As a result, we conclude that the vertical strip-like features shown in the FIM result from the dendritic structure in the epitaxial cladding layers. The dark lines are the deformed inter-dendrite subgrain boundaries, while the bright regions are the intra-dendrite regions of good single crystallinity. To cross check this conclusion, high magnification BSE image is taken in the epitaxially deposited region, and shown in Fig. 5c. Inter-dendrite boundaries are visible in the region, highlighted by high concentration of tiny precipitates with high brightness. The dendrite widths are measured to be 3–6 μm in this image, agreeing well with the FIM observation.

μXRD has been proven to be an important technique to study the microstructures of metals, alloys, ceramics, and minerals. To explore its application, here we introduce a novel data analysis approach by calculating the average intensity with or without filtering to realize real-time microstructure imaging. By this means, it is easy to analyze ten or more Laue patterns per second on an average desktop or laptop computer, with the processing speed mainly depending on the reading/writing speed of the hard disk drive. Because it is not necessary to index the patterns, the analysis speed will not slow down for materials with poor crystallinity, complicated crystal structure, or with multiple phases. Compared with a typical μXRD experiment, the time consumed by this analysis method is almost negligible, and thus the microstructures of the scanned material can be visualized in real time. For example, in the 3D printed Ni-based superalloy experiment, described above, the 13260-step raster scan took about 4 hours to finish, the speed being mainly limited by the motion of the sample stages. Data analysis time on a personal computer could vary greatly, depending on the specific experiment-dependent parameters used such as crystal structure, but in average took a day to obtain a reasonable 2D mapping of a multi-phase polycrystalline sample. By using the approach introduced here, the intensity maps were readily generated within 1 hour on a laptop computer, the speed being mostly determined by how fast the Laue frames could be read on the hard disk, while the time needed for computing the intensity was almost negligible. However, there are a few caveats to this analytical approach. First of all, it does not help improve the resolution of the μXRD technique; therefore, features beyond the spatial resolution of μXRD are invisible, like the tiny precipitates concentrated at the subgrain boundaries in the cladding region shown in this article. Secondly, although this method provides a quick view of the microstructures, we expect that in some cases it is still necessary to check the local diffraction patterns for a better understanding of the characteristic features in the microstructural maps. For example, to measure crystal orientation, elastic strain distribution^32^, to distinguish between statistically stored dislocations (SSDs) and geometrically necessary dislocations (GNDs)^33^, to characterize the slip system of the GNDs^34^, to quantify dislocation densities^35^, one still needs to index the Laue pattern and analyze peak shape the usual way. Furthermore, although we demonstrate that the low angle grain boundaries are visible in the FIM, it is noted that from the FIM solely, it is difficult to distinguish between phase boundaries, low angle grain boundaries, high angle grain boundaries, and twin boundaries, and therefore it is necessary to index the Laue patterns^36^. An example containing both low and high angle grain boundaries is shown in Section 2 of the supplementary information.

In summary, in this article we study the microstructure of a 3D printed Ni-based superalloy using synchrotron-based μXRD Laue technique. A novel analytical approach is employed. The HAZ region with high dislocation density is suggested by the streaking of Laue peaks. A crack is observed to propagate along a high angle grain boundary, surrounded by a plastically deformed region. No coherency relationship are found between the micron-sized MC carbide precipitates and the Ni-matrix, which may have an essential impact on the mechanical properties of the superalloy. The morphology and size of the dendritic microstructures in the epitaxial region are visualized from the contrast resulting from the intergranular low angle grain boundaries. The combination of the μXRD technique and the newly developed analytical method ensures a prompt and thorough characterization of almost all the important aspects of Ni-based superalloys, and may provide new opportunities to explore applications of the μXRD technique to a range of problems in material and geosciences. The newly developed analytical approach provides a quick overview of all microstructural characteristics of the sample before a more detailed study can be conducted for each of the observed features, and thus is an important addition to the arsenal of tools used for interpreting μXRD scans.

## Methods

### Laser assisted 3D printing

The 0.8 mm thin-wall substrate was cut from a directionally solidified DZ125L Ni-based superalloy. The laser assisted 3D printing was conducted using an independently developed XJTU-1 3D printing system, equipped with a Nd:YAG laser with a beam size of 0.5 mm^37^. During the cladding process, powder with spherical shape particles, 50–100 μm in diameter, and similar chemical composition to the substrate, was injected at a 9 mm^3^/s feeding speed by Ar gas carrier to the molten pool, generated by laser heating with a power of 230 W and 4 mm/s laser scanning speed on the (100) crystal plane of the substrate. Consequently, the molten powder was deposited at the top of the substrate, layer by layer during several passes of the scanning laser. The chemical composition of the DZ125L Ni-based superalloy substrate and of the powder can be found elsewhere^19^,^20^.

### μXRD study

The mirror finish of **X-Y** cross-section plane on the 3D printed sample was obtained via traditional mechanical polishing procedure, and an area which was close to the substrate-cladding interface and a crack and some precipitates were visible was marked under an optical microscope (OM). The μXRD measurement of this area was carried out on Beamline 12.3.2 at the Advanced Light Source (ALS) of the Lawrence Berkeley National Laboratory (LBNL)^38^. In this technique, a synchrotron polychromatic X-ray beam with a continuous spectrum between 5 and 24 keV was focused to a spot size of about 1 × 1 μm^2^ using a pair of orthogonal Kirkpatrick-Baez mirrors. The laser 3D printed sample was mounted on a high resolution **X**-**Y** scanning stage that was tilted 45° relative to the incident X-ray beam. The vertical **Y**-scanning direction was perpendicular to the substrate-cladding interface, while the horizontal **X**-direction was normal to **Y** but also within the sample surface plane. A 130 μm (horizontal) × 408 μm (vertical) area was scanned with a fixed scanning step size of 2 μm, resulting in a total of 13260 steps in the scan. At each scanning position, the X-ray penetrated about 40 μm and a Laue diffraction pattern was recorded in reflection mode with a 2D DECTRIS Pilatus-1M detector, mounted approximately 150 mm above the probe spot and 90° with respect to the incoming X-ray. At each scanning position the exposure time to obtain a diffraction pattern was 1 s.

### SEM observation

After the μXRD experiment, the same area of the 3D printed sample was investigated under BSE mode in a Hitachi SU6600 field emission SEM operating at a 10 kV accelerating voltage. Under these conditions, most of the BSE signals were collected within hundreds of nm underneath the sample surface according to the simulation using CASINO^39^. The crack and precipitates were clearly observed, and slight contrast was also seen at the substrate-cladding interface.

### WDS characterization of the precipitates

The element distribution of the precipitates was characterized via WDS in a XA-8230 SuperProbe Electron Probe Microanalyzer. The accelerating voltage was 20 kV in this study. The element concentration distributions of Ni, Co, Cr, Al, Ta, Ti, W, and C were mapped.

## Additional Information

**How to cite this article**: Zhou, G. *et al.* Real-time microstructure imaging by Laue microdiffraction: A sample application in laser 3D printed Ni-based superalloys. *Sci. Rep.* **6**, 28144; doi: 10.1038/srep28144 (2016).

## Supplementary Material

Supplementary Information
