# Supplementary material for: Real-time microstructure imaging by Laue microdiffraction: A sample application in laser 3D printed Ni-based superalloys
Source: Sci Rep. 2016 Jun 15;6:28144. doi: 10.1038/srep28144 (PMC4908403; doi:10.1038/srep28144)
Supplement: Supplementary Information [file srep28144-s1.pdf]

Real-time microstructure imaging by Laue microdiffraction: A sample application in laser 3D printed Ni-based superalloys

Guangni Zhou[1*], Wenxin Zhu[1*], Hao Shen[1], Yao Li[1], Anfeng Zhang[2],

Nobumichi Tamura[3], Kai Chen[1]

1. Center for Advancing Materials Performance from the Nanoscale (CAMP-Nano), State Key Laboratory for Mechanical Behavior of Materials, Xi'an Jiaotong University, Xi'an, Shaanxi 710049, P.R. China
2. State Key Laboratory for Manufacturing Systems Engineering, Xi'an Jiaotong University, Xi'an, Shaanxi 710049, P.R. China
3. Advanced Light Source, Lawrence Berkeley National Laboratory, Berkeley, California 94720, USA

* These authors contributed equally to this work.

Correspondence should be addressed to K.C. (email: kchenlbl@gmail.com)

1. The FIM plotted following Option 1

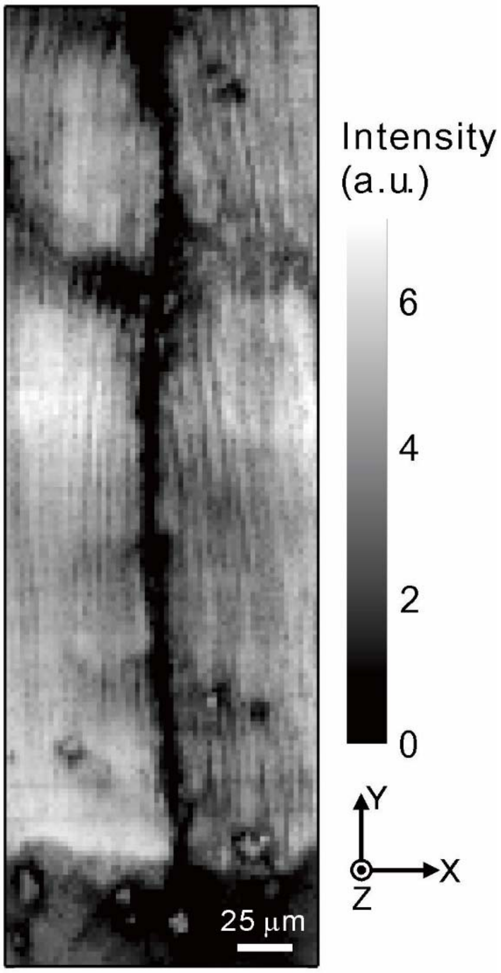

Figure S1 | The FIM plotted following Option 1 as described in the main text. This map shows almost identical features and contrast to the FIM shown in the main text, which is plotted following Option 2.

To compare these two options described in the main text, the pros and cons of either method are concluded. The physical meaning of the average filtered intensity calculated with Option 1 is clear, and we expect that this approach is universal to almost all crystalline samples. Option 2 is even faster than Option 1 because the background intensity is not fitted; however, because the fluorescence signal intensity is usually higher in the center of the detector than in the edge, more intensities will be

subtracted from the peaks in the edge than from the peaks in the center, therefore the averaged filtered intensity computed in this approach is meaningful only qualitatively.

2. Distinguish of high and low angle grain boundaries from the Filtered Intensity Map (FIM)

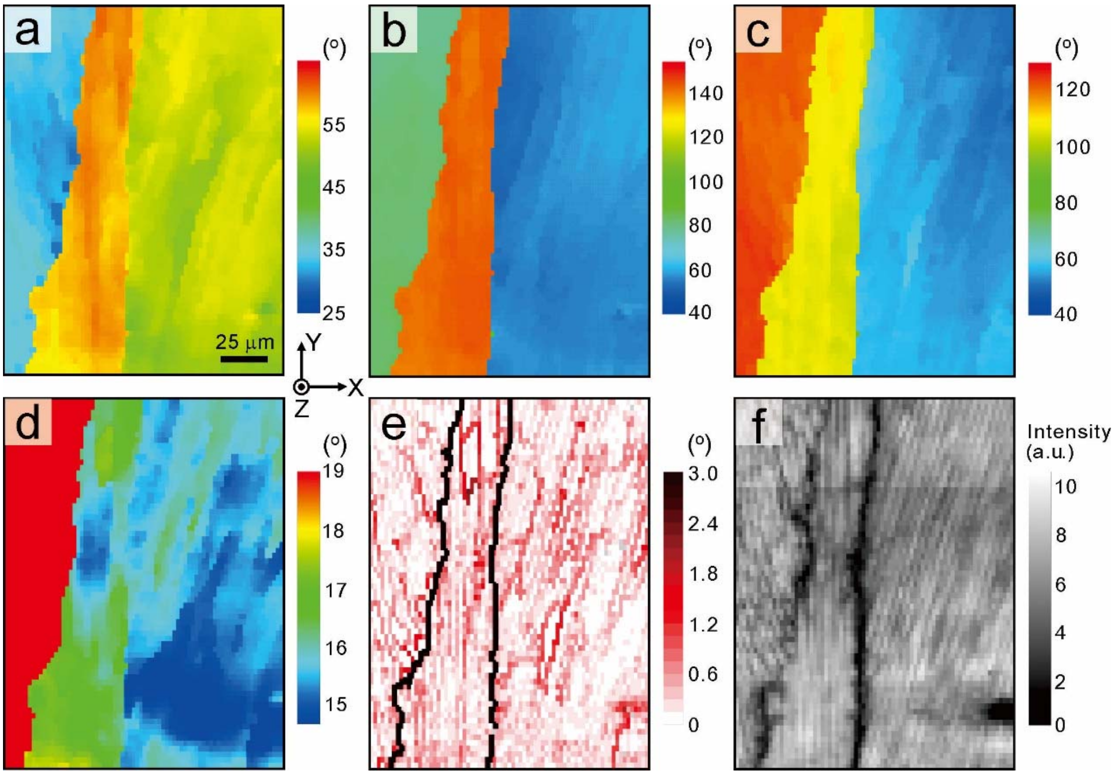

Figure S2 | Characterization of a 3D printed Ni-based superalloy specimen that contains high angle grain boundaries. The crystal orientation of the scanned area is demonstrated in rotation axis-angle representation. (a-c) The angles between the rotation axis and the **X**-, **Y**- and **Z**-axis are displayed in a-c, respectively, and (d) the rotation angle is shown in d. (e) The disorientation distribution is computed and plotted, where high and low angle grain boundaries are visible. (f) With the method introduced in the main text, the FIM is charted, in which high and low angle grain boundaries are displayed as low intensity.

As shown in the main text, low angle grain boundaries are visible from the FIM. As a test, a 150 × 201 μm^2 area in an identified 3D printed Ni-based superalloy is scanned with the μXRD technique, and the scanning step size is 2 μm horizontally and 3 μm vertically. The data are analyzed in the similar way as introduced in the

main text, as well as the conventional approach. The crystal orientation distribution in the scanned area is represented using the rotation axis-angle notation. Fig. S2a-c show the angles between the rotation axis and the **X**-, **Y**-, and **Z**-axis, respectively, and Fig. S2d shows the rotation angle distribution. The disorientation angle distribution is computed and displayed in Fig. S2e, showing two high angle grain boundaries (>5°) as well as low angle subgrain boundaries. In the FIM shown in Fig. S2f, both high and low angle grain boundaries are visible. In this case, the high angle grain boundaries look darker and thicker than the low angle ones; however, a higher angle grain boundary does not necessarily give lower intensity. Therefore, it is not so easy to tell the type of a boundary unambiguously if no further information is provided, or if the Laue patterns are not indexed.
